# Chestnut Drying Is Critical in Determining *Aspergillus flavus* Growth and Aflatoxin Contamination

**DOI:** 10.3390/toxins10120530

**Published:** 2018-12-11

**Authors:** Simona Prencipe, Ilenia Siciliano, Carlotta Gatti, Maria Lodovica Gullino, Angelo Garibaldi, Davide Spadaro

**Affiliations:** 1 DISAFA—Dipartimento di Scienze Agrarie, Forestali ed Alimentari, Università degli Studi di Torino, Largo P. Braccini 2, 10095 Grugliasco, Italy; simona.prencipe@unito.it (S.P.); carlottagatti.6@gmail.com (C.G.); marialodovica.gullino@unito.it (M.L.G.); 2 Centro di Competenza per l’Innovazione in Campo Agro-ambientale (AGROINNOVA), Università degli Studi di Torino, Largo P. Braccini 2, 10095 Grugliasco, Italy; ilenia.siciliano@unito.it (I.S.); angelo.garibaldi@unito.it (A.G.)

**Keywords:** aflatoxins, antioxidant activity, *Castanea sativa*, composition, temperature, total phenol content

## Abstract

Chestnut drying is used to prevent postharvest losses and microorganism contamination during storage. Several studies reported the contamination by aflatoxins (AFs) produced by *Aspergillus* spp. in chestnuts. The effect of drying temperatures (from 30 to 50 °C) was evaluated on the growth of *A. flavus* and the production of aflatoxins in chestnuts. The influence of the treatment on the proximate composition, the total phenol content and antioxidant activity of chestnuts was considered. Fungal colonization was observed on the nuts dried at 30, 35, and 40 °C; the incidence was lower at 40 °C. The highest concentrations of AFB_1_ and AFB_2_ were produced at 40 °C. No aflatoxins were detected at 45 or 50 °C. At 40 °C *A. flavus* was under suboptimal conditions for growth (*a_w_* 0.78), but the fungus was able to synthesize aflatoxins. As the temperatures applied increased, the total phenol content increased, while the antioxidant activity decreased. A drying treatment at 45 °C for seven days (*a_w_* 0.64) could be a promising method to effectively control both the growth of aflatoxigenic fungi and the production of aflatoxins. This study provides preliminary data useful to improve the current drying conditions used in chestnut mills, to reduce both fungal growth and aflatoxin production.

## 1. Introduction

Chestnuts are produced in different countries worldwide, particularly in Asia and Europe, but also in the United States, Australia, New Zealand and Chile [1]. Approximately 20% of the chestnuts are used for the industrial preparation of chestnut flour, dried chestnuts and marron glacés [2].

Chestnuts have interesting nutritional characteristics, as they are rich in carbohydrates (around 40%), including starch, they contain interesting minerals, vitamins and relevant levels of fiber, but low amounts of proteins (2–4%) and fats (0.5–5%) [3,4,5,6]. They are also an interesting source of essential fatty acids [7]. Chestnuts have a high water (around 50%; [3]) and sugar content that limits the postharvest life [8]. Researchers focused on the implementation of effective methods to increase the chestnut storage life, while preserving their chemical composition [9,10].

Different species of *Fusarium*, *Penicillium* and, *Aspergillus* have been isolated from chestnuts during postharvest [11,12]. Several *Penicillium* and *Aspergillus* species, originating from chestnuts, showed to be able to produce mycotoxins, such as aflatoxins, ochratoxin A, citrinin, roquefortine C and mycophenolic acid in chestnut derived products [2,13].

*Aspergillus* section *Flavi*, and in particular *A. flavus* and *A. parasiticus*, produce aflatoxins (AFs), a group of mutagenic, teratogenic and immunosuppressive mycotoxins [14]. The most common aflatoxins (AFs) are aflatoxin B_1_ (AFB_1_), aflatoxin B_2_ (AFB_2_), aflatoxin G_1_ (AFG_1_) and aflatoxin G_2_ (AFG_2_) [15]. AFB_1_ is genotoxic, it causes liver cancer in most animal species, and it shows the strongest carcinogenicity among all the aflatoxins [16,17]. *A. flavus* produces AFB_1_ and AFB_2_, while *A. parasiticus* produces also AFG_1_ and AFG_2_. In particular, the occurrence of *Aspergillus* section *Flavi* can lead to important economic losses, and their growth depends on several factors, such as product composition, pH, water activity (*a_w_*), temperature and storage time [18]. Different strategies could be adopted in order to reduce AFs in foodstuff [19], including the use of chemical and biological control in the field or the application of different post-harvest treatments, like sorting procedures, thermal treatment, γ irradiation, ozone fumigation and cold plasma [20,21,22,23].

In recent years, the occurrence of aflatoxins in chestnut products has been reported with increasing frequency [24,25]. Recently, a monitoring of *Aspergillus* section *Flavi* and their potential role in aflatoxin production was performed on chestnuts and their derivatives [26]. The European Commission (EC) has set the maximum limits for aflatoxins in different foodstuffs [27] with the Regulation 165/2010 that fixed the maximum levels of AFB_1_ and total AFs at 2.0 and 4.0 µg/kg, respectively, for some nuts, including chestnuts.

Drying of agricultural products is a technique that is commonly used to prevent fungal growth [12,28]. Some studies explored the effect of environmental parameters on *A. flavus* growth and AFs production in different matrices [29,30], but no studies have focused on the effect of the drying temperatures on *A. flavus* growth and AFs production. Previously, Attanasio et al. [8] showed that different drying temperatures could affect the physico-chemical properties of chestnuts.

Considering the possible contamination of chestnut products by aflatoxins, the present study reports the effect of different drying temperatures on the growth of *A. flavus* and the production of aflatoxins in chestnuts to be processed in the mills for chestnut flour and granulate production. Furthermore, some quality and chemical parameters of the fruits were considered, in order to evaluate the effect of the drying treatments on the proximate composition, the total phenol content and antioxidant activity of chestnuts.

## 2. Results

### 2.1. A. flavus Growth at Different Temperatures

Fungal colonization was observed after seven days of treatment and after seven days of storage on all the samples dried at 30, 35, and 40 °C. After seven days treatment, the incidence was 90.8%, 85.0% and 10.0% at 30, 35 and 40 °C, respectively. No fungal growth was observed for chestnuts treated at 45 °C and 50 °C (data not shown).

**Figure 1:**
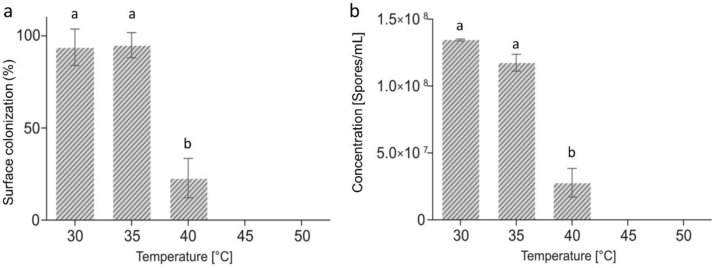
Fungal colonization. (**a**) Percentage (%) of chestnut surface colonized by *Aspergillus flavus* and (**b**) concentration of viable *A. flavus* spores on chestnuts after treatment from 30 °C to 50 °C for seven days and seven days of shelf life at 22 °C. Values are expressed as mean values ± SD (*n* = 12 per experiment; two repetitions of the experiments). Values followed by the same letter are not statistically different by Duncan’s multiple range test (*p* < 0.05).

After seven days storage at 30 °C and 35 °C, the fungal colonization was not significantly different, with incidences of 93.8% and 94.9%, compared to the incidence after the treatment. The incidence was lower at 40 °C (22.9%), but significantly higher compared to the incidence after seven days of thermal treatment. At 45 °C and 50 °C, no fungal growth was observed (Figure 1a.).

No significant differences were found for the number of spores of *A. flavus,* with 1.35 × 10^8^ spores/mL and 1.17 × 10^8^ spores/mL for the samples treated at 30 °C and 35 °C, respectively (Figure 1b). At 40 °C, the concentration was 2.77 × 10^7^ spores/mL. No spores were viable on the chestnuts treated at 45 °C or 50 °C.

### 2.2. Aflatoxin analysis

**Figure 2:**
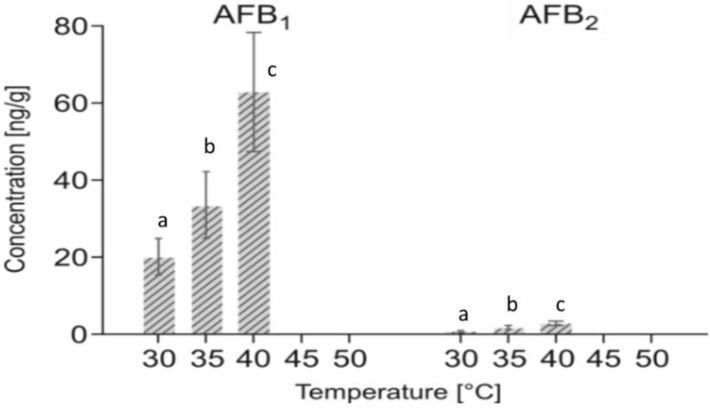
Concentrations (ng/g) of AFB_1_ and AFB_2_ on chestnuts treated at different temperatures from 30 °C to 50 °C for seven days and seven days of shelf life at 22 °C. Values are expressed as mean values ± SD (*n* = 12 per experiment; two repetitions of the experiments). Values followed by the same letter are not statistically different by Duncan’s multiple range test (*p* < 0.05).

The concentrations of AFB_1_, AFB_2_, AFG_1_, and AFG_2_ was investigated for each temperature. AFG_1_ and AFG_2_ were not detected in any of the tested conditions. The highest concentrations of AFB_1_ (62.92 ng/g) and AFB_2_ (2.87 ng/g) (Figure 2.) were produced at 40 °C, while the concentrations of AFB_1_ were 20.15 ng/g and 33.60 ng/g at 30 °C and 35 °C, respectively, while the concentrations of AFB_2_ were 0.72 ng/g and 1.81 ng/g, respectively. No aflatoxins were detected at 45 °C or 50 °C.

### 2.3. Composition

The chemical composition of the chestnuts is reported in Table 1. The moisture content (%) decreased from harvest to the treatment with the highest temperature. Significant differences were found in the moisture content between raw chestnuts and chestnuts subjected to drying. Chestnuts treated at 30 °C had a reduction of 41% in the moisture content compared to the untreated fruits. Significant differences were found between chestnuts treated at 35 °C and 40 °C, with a reduction of moisture content of 56% and 64%, respectively. The treatments at 45 °C and 50 °C further decreased the moisture content. Besides, the water activity was measured on the samples at harvest (*a_w_* 0.96) and after the thermal treatments. The values decreased with increasing temperatures, from *a_w_* 0.92 at 30 °C, 0.83 at 35 °C, 0.78 at 40 °C, 0.64 at 45 °C, and 0.36 at 50 °C.

The fat content increased for the highest temperature, with significant differences. Carbohydrates increased with the temperature of the treatment and were statistically different, with values ranging from 62.23 g/100 g to 77.90 g/100 g. No statistical differences were found for fiber or protein contents, with mean values of 5.68 g/100 g for the fiber content and 3.53 g/100 g for protein.

### 2.4. Total Phenol Content (TPC) and Antioxidant Activity

The total phenol content increased as the temperatures applied increased (Table 2). At harvest, TPC was 60.8 mg gallic acid equivalent (GAE)/100 g fresh weight (FW). Statistical differences were found for the treatments from 35 °C to 50 °C, with 66.2 mg GAE/100 g FW, 74.3 mg GAE/100 g FW, 92.3 mg GAE/100 g FW, and 131.7 mg GAE/100 g FW, for 35 °C, 40 °C, 45 °C, and 50 °C respectively.

The antioxidant activity decreased as the temperatures applied increased (Table 2). At harvest, the antioxidant activity was 928 µmol ascorbic acid equivalent (AAE)/100 g FW. Statistical differences were found for the treatments at 45 °C, with 665 µmol AAE/100 g FW, and at 50 °C, with 644 µmol AAE/100 g FW.

## 3. Discussion

Aflatoxin contamination of chestnut products has been reported in different studies. Pietri and colleagues [24] showed an incidence of AFs contamination close to 60%, and 20% of the samples exceeded the European limits, with 62.2% AFB_1_ for chestnut flour and 21.4% for dried chestnuts. Bertuzzi et al. [2] found AFs contamination in fresh chestnuts and dried chestnuts, with an incidence of 15% and 40%, respectively, while a higher incidence (92%) was found for chestnut flour samples, with 24% exceeding the EU limits for AFB_1_. The European Rapid Alert System for Food and Feed [25] also reported several AFs contaminations in dried chestnuts, chestnut flakes and chestnut flour produced in Italy. Because of their characteristics (high moisture, starch, and water contents), chestnuts are more susceptible to the growth of mycotoxigenic fungi, especially during storage [11,12]. This scenario underlines the problems related to AFs in meeting the EU legislative limits concerning food safety.

Several studies have shown that temperature and water activity have a great effect on *A. flavus* growth and aflatoxin production [31,32,33]. Different methods have been successfully applied to chestnuts in order to reduce fungal growth and prolong shelf life [28,34,35].

In our study, the effect of different drying temperatures on both *A. flavus* growth and AFs production was evaluated in order to reduce the risk of aflatoxin contamination of chestnuts. After drying, the fruits were kept at 22 °C for seven days in order to simulate the shelf life conditions of the products.

As reported by Shindler et al. [36], the optimal growth temperatures for *A. flavus* is not the same as those for aflatoxin production. The optimal temperature for growth of *A. flavus* is 30–33 °C [37], and our data have confirmed the literature results, with the highest concentration of *A. flavus* found in the chestnuts dried at 30 °C and 35 °C. At these temperatures, the measured *a_w_* was higher than the minimum *a_w_* permitting the growth of *A. flavus* [38], and the results confirmed the ability of the fungus to grow. We could affirm that a combined effect of the temperature and the *a_w_* was able to favour the fungal colonization.

A lower growth of *A. flavus* was found at 40 °C treatment and seven days of shelf life. After the 40 °C treatment, the final *a*_w_ was 0.78, which is close to the minimum *a_w_* permitting the growth of *A. flavus.* At this temperature, the AFB_1_ and AFB_2_ concentrations were the highest ones. At 40 °C, *A. flavus* was under suboptimal conditions for growth, but after the following seven days at 22 °C the incidence of *A. flavus* increased and the fungus was able to produce aflatoxins. Similarly, Lahouar et al. [39] found the highest amount of AFB_1_ on sorghum at about 37 °C, while Liu et al. [30] reported a decrease of AFs concentration in peanuts at 37 °C. On the contrary, the highest AF contamination in maize and soybean has been shown to occur at 30–35 °C [40,41].

Liu and colleagues [30] reported that a drying temperature of above 42 °C could be suppressive for aflatoxin biosynthesis and growth of *A. flavus* on shelled peanuts. Similarly, no mold growth was observed on inoculated chestnuts treated at 45 °C or 50 °C followed by shelf life for seven days, and aflatoxins were not detected. Furthermore, the maximum temperature for *A. flavus* growth, as reported by Pitt and Hocking [37], was around 43–48 °C. Similar results were shown by Marín et al. [29], who used predictive models on pistachio: after 15 days of incubation, the concentration of aflatoxins increased with increasing the applied temperatures. It started to decrease at 37 °C, while no AFs were detected at 42 °C.

In commercial chestnut mills, fresh chestnuts are dried as soon as they reach the mill, and afterwards they undergo sorting, roasting, granulation, milling, packaging, and storage until sale [26].

The current conditions for drying chestnuts before processing include a drying under air flow at 30 °C for three to five days, until they reach 10% humidity. The results obtained in this work show that seven days at 30 °C in static air was not enough to reach 10% humidity, and temperature higher than 40 °C was needed to reach values of residual humidity close to 10%.

When considering the effect of the applied thermal treatments on quality, some changes in the chemical composition of the chestnuts were observed. As reported by Goncalves et al. [42], boiling and roasting treatments affected the quality of chestnuts.

The effect of drying in non-inoculated chestnuts reduced the moisture content from 45% to 9%, and an increase in the carbohydrate and fat contents, compared to the raw chestnuts, was observed for the treatment at the highest temperatures. A higher fat content was also observed for boiled chestnut [42], while an increase in reducing sugars was observed by Correira and colleagues [43]. No effect was observed on the fiber or protein content. The cooking processes significantly affected the composition of the chestnuts due to the water losses that consequently increased the concentration of the other compounds, such as carbohydrates.

Previous data on the total phenol content and antioxidant activity of chestnuts mainly refer to fresh nuts [44,45,46]. Limited data have been reported on the effects of cooking processes and of drying temperatures on these parameters [35].

The TPC analysis revealed an increase in water extractable phenol content as the applied temperature was increased, and similar results were obtained by Barros et al. [47] and Goncalves et al. [42] in their analyses of boiled and roasted chestnuts. Polyphenols are substances that are free in the chestnuts tissues and not strongly bound to the fruit structure, due to the water diffusion [10]. Probably, the extraction of TPC in water increased the recovery of these compounds [35].

The antioxidant activity was reduced by increasing the applied temperature, but significant differences were only found at 45 °C and 50 °C. Similar results about the antioxidant activity were found for roasting chestnuts of Longal2 and Trigueira varieties, compared to raw chestnuts [47], as well as for chestnuts subjected to roasting, boiling and curing [10]. As reported by Ribeiro et al. [48], heat is the main cause of modification of the organic acids’ contents of chestnuts samples, due to the reduction of citric and ascorbic acids. Furthermore, in the study of Barros and colleagues [46], a positive correlation between a higher TPC and a lower antioxidant capacity was found only for one cultivar over nine analyzed, with a higher amount of gallic acid. These findings could be attributed to the cultivar analysed as reported by Zhu [35].

In conclusion, the present study showed that a drying treatment at 45 °C for seven days could be a promising method to effectively control both the growth of aflatoxigenic fungi and the production of aflatoxins, due to the low moisture content and *a*_w_ reached. A study about the kinetics of the *a_w_* and water content could be performed in drying facilities for chestnuts, to reduce the length of the treatment. After a treatment at 45 °C, only minor changes occurred in the chemical composition of the chestnuts, and good levels of fatty acids, phenols, proteins, and fibers were preserved. Furthermore, chestnuts are mostly consumed after cooking (boiling or roasting), and in our analyses the chemical changes that occurred were similar to those found after cooking chestnuts. This study provides preliminary data useful to improve the current drying conditions used in chestnut mills, to reduce both fungal growth and aflatoxins production. The increase of temperature until 45 °C for chestnut drying before processing, could prevent *A. flavus* growth and AFs contamination, without affecting the overall quality of the chestnuts. Further studies should be accomplished to apply the drying conditions in commercial chestnut mills. Drying should be considered a prevention practice to avoid aflatoxin contamination, and thus to avoid production and economic losses.

## 4. Materials and Methods

### 4.1. Fungal Strain

A strain of *Aspergillus flavus*, named AFSP4, isolated from chestnut and able to produce both aflatoxins B_1_ and B_2_ was used to inoculate chestnuts during the experiments [26]. The strain was grown on Potato Dextrose Agar (PDA, Merck, Darmstadt, Germany) at 30 °C for seven days. In order to collect the spores, 5 mL of sterile deionized water, containing 1% Tween, was added to each plate and the colony surface was gently scraped. The resulting spore suspension was counted using a Bürker chamber to obtain a concentration of 1 × 10^6^ spores/mL.

### 4.2. Inoculation of Chestnuts and Fungal Spore Count

Freshly harvested chestnuts (*Castanea sativa* Mill. var. Gabiana), used for fungal inoculation and aflatoxin analysis, were harvested in the Cuneo province (northwestern Italy) in September 2016 and they were stored in sealed polypropylene plastic bags at 4 °C until used for the experiments.

The chestnuts were sterilized with 1% sodium hypochlorite, washed with sterile deionized water and allowed to air dry. Three wounds were made per chestnut (1 cm long), and 500 μL of the fungal spore suspension were inoculated in each wound. Similarly, 120 control fruits were inoculated with sterile deionized water. After inoculation, 120 chestnuts were stored in perforated plastic boxes (eight chestnuts per box, three replicates per temperature, 5 temperatures), at 30 ± 1 °C, 35 ± 1 °C, 40 ± 1 °C, 45 ± 1 °C or 50 ± 1 °C for seven days, and at room temperature (22 ± 1 °C) for seven days to monitor fungal growth. The experiment was performed twice.

In order to assess the fungal contamination, each chestnut was visually examined, after seven days treatment and, a second time, after seven days storage, to give an index of surface colonization: 0 (from 0% to 5%); 1 (from 6% to 25%); 2 (from 26% to 50%); 3 (from 51% to 75%); 4 (from 76% to 90%); 5 (91% to 100%). The average index was transformed into percentage with 100% corresponding to index 5. Afterwards, four chestnuts per box were placed in a beaker (500 mL) with 50 mL of sterile water containing 1% Tween (Merck); the beaker was then placed on a rotary shaker for 60 min. The samples were diluted and *A. flavus* spores were counted using a Bürker chamber and plated on potato dextrose agar (100 µL/plate). After 48 h of incubation at 30 °C, the fungal colonies were visually counted on plates in order to assess the viability.

### 4.3. Aflatoxin Extraction and Analysis

Aflatoxin production was determined after seven days of treatment at different temperatures followed by seven days of storage at 22 °C. The remaining four chestnuts per box were weighed individually, crumbled, and placed in a Falcon tube (50 mL); 20 mL of acetone was then added and aflatoxins were extracted using a rotary-shaking stirrer for 30 min. The extract was transferred into a Falcon tube and centrifuged for 2 min. The extract was evaporated until dryness and recovered with 500 μL of water:acetonitrile 50:50. Each biological replicate (three replicates) was composed of four chestnuts and the AFs were extracted from four chestnuts (technical replicates). The experiment was performed twice. The same procedure was also applied to the uninoculated samples, which resulted free of AFs.

Aflatoxin analysis was performed using a Varian Model 212-LC micro pump (Varian Medical Systems Inc., Palo Alto, CA, USA) with a Varian autosampler Model 410 Prostar coupled with a Varian 310-MS triple quadrupole mass spectrometer and with an electrospray ion source (ESI) operating in positive ionization mode. Chromatographic separation was performed in isocratic mode on a Pursuit XRs Ultra C18 (100 mm × 2.0 mm, 2.8 μm, Varian) column, using water acidified with 0.05% of formic acid (Sigma-Aldrich, St. Louis, MO, USA) and methanol (Merck) (40:60 *v*/*v*) as eluents; the flow rate was set at 0.2 mL/min for 10 min. The monitoring reaction mode transitions used for quantification were: 313 > 285 (CE 14 V) for AFB_1_ and 315 > 287 (CE 18 V) for AFB_2_.

In order to quantify the AFs content in the samples, the external standard method was used. A standard curve with a mixture of AFB_1_ and AFB_2_ standards was built, using concentrations ranging from 1 to 500 ng/mL. Extraction recovery was evaluated using chestnuts not heat-treated and contaminated with the standards of AFB1 and AFB2. Chestnuts were contaminated before extraction procedure at two concentrations (5 and 25 ng/g). Three replicates were prepared for each concentration and the average results obtained for AFB1 and AFB2 were 72.4 ± 1.3% and 72.1 ± 1.7% respectively.

### 4.4. Chestnut Composition

Chestnuts (three technical and three biological replicates) were analyzed at harvest, and after each treatment for moisture content (%), fiber, fat, carbohydrate and protein contents (g/100 g), by Agrobiolab (Rutigliano, Italy).

The moisture content was determined after drying in air oven at 103 °C (ISTISAN 1996/34 p. 7, method B, [49]). The total protein content was obtained according to the Kjeldahl method (ISTISAN 96/34 p. 13, [49]). Total dietary fiber quantification was performed according to 985.29 AOAC method [50]. Total fat content was determined by using the Soxhlet extraction method (AOAC 920.39, ether extraction [51]) and gas chromatography-mass spectrometry. The carbohydrates were determined by HPLC, after clarification with Carrez reagent (ISTISAN 1996/34 p. 63, [49]).

Besides, the water activity (*a_w_*) was measured on the chestnuts treated or not by using an electronic hygrometer AquaLab Series 3TE (Decagon Devices Inc., Pullman, WA, USA), which adopted the chilled-mirror technique at 25 °C.

### 4.5. Total Phenol Content (TPC) and Antioxidant Activity

Chestnuts (three technical and three biological replicates) were analyzed at harvest, and after each thermal treatment for water extractable TPC and antioxidant capacity. Chestnuts were peeled, weighed and transferred to a falcon tube (50 mL). Extraction was performed by shaking the chestnuts at 1400 rpm for 1 h in deionized water, with a solid/liquid ratio of 1/10. Samples were centrifuged at 10,000× *g* for 10 min and the supernatant was collected. The solid fraction was further extracted and the two supernatants were combined. The extracts were immediately analyzed to establish the total phenolic content and the antioxidant activity.

The Folin-Ciocalteu method [52] was used to analyze the TPC content using: 0.5 mL of the aqueous solution of the extract, 2.5 mL of the Folin-Ciocalteu reactive (diluted 1:10, *v*/*v*) and 2 mL of 75 g/L Na_2_CO_3_ aqueous solution. The samples were incubated at 50 °C and, after 5 min, the absorbance was measured at 760 nm. Gallic acid was used as the standard and the TPC content was expressed as mg gallic acid equivalent (GAE) per 100 g extract, in fresh weight (FW).

The ferric reducing antioxidant power (FRAP) assay was used to evaluate the antioxidant activity, according to Szöllösi and Szöllösi-Varga, [53]. Briefly, 100 µL of the aqueous solution of the extract was added to 300 mL of a fresh FRAP reagent (25 mL acetate buffer, 300 nmol/L, pH 3.6; 2.5 mL of TPTZ (2,4,6-tripyridyl-1,3,5-triazine) diluted in 40 nmol/L HCl and 2.5 mL of 20 nmol/L FeCl_3_⋅6H2O was added. After 5 min, the absorbance was measured at 593 nm. Ascorbic acid equivalent (AAE) was used as the standard, and the results were expressed as µmol AAE per 100 g extract in fresh weight (FW).

### 4.6. Statistical Analysis

Statistical analyses on the percentage of chestnut surface colonized by *A. flavus,* concentration of viable *A. flavus* spores, aflatoxin production, chestnut composition, total phenol content and antioxidant activity were performed using IBM SPSS statistics software Inc. version 22 (Chicago, IL, USA), for variance analysis (one-way analysis of variance) using the Duncan test with *p* ≤ 0.05.

## Figures and Tables

**Table 1:** Average moisture content (%), fiber (g/100 g), fat content (g/100 g), carbohydrates (g/100 g) and protein (g/100 g) for raw chestnuts at harvest, and treated at different temperatures (from 30 to 50 °C) for 7 days.

Sample	Moisture [%] *			Fiber [g/100 g] *			Fat Content [g/100 g] *			Carbohydrates [g/100 g] *			Protein [g/100 g] *		
Harvest (Raw)	45.50	±0.46	a	6.51	±0.10	a	0.47	±0.02	a	42.65	±0.06	a	3.51	±0.08	a
Treatment 30 °C	26.67	±1.35	b	5.25	±0.35	a	0.56	±0.09	a	62.23	±2.15	b	3.53	±0.05	a
Treatment 35 °C	19.87	±1.45	c	5.23	±1.21	a	0.56	±0.03	a	70.00	±1.20	c	3.46	±0.03	a
Treatment 40 °C	16.30	±1.60	c	5.32	±0.23	a	0.61	±0.05	ab	72.65	±4.25	c	3.51	±0.08	a
Treatment 45 °C	11.30	±1.40	d	5.42	±1.15	a	0.71	±0.03	b	77.35	±0.55	d	3.50	±0.08	a
Treatment 50 °C	9.62	±1.48	d	5.51	±0.32	a	0.91	±0.03	c	77.90	±1.39	d	3.66	±0.18	a

* Values are expressed as mean value ± SD (*n* = 9). Values followed by the same letter are not statistically different by Duncan’s multiple range test (*p* < 0.05).

**Table 2:** Average total phenol content (mg GAE/100 g extract in fresh weight (FW)) and antioxidant activity (µmol AAE/100 g extract in FW) of raw chestnuts at harvest, and after treatment at different temperatures (from 30 to 50 °C).

Treatment	Total Phenol Content [mg GAE */100 g FW] *		Antioxidant Activity [µmol AAE **/100 g FW] **	
Harvest (raw)	60.8 ± 2.02	a	928 ± 1.9	c
Treatment 30 °C	63.3 ± 3.44	ab	884 ± 1.5	bc
Treatment 35 °C	66.2 ± 6.18	b	755 ± 1.3	ac
Treatment 40 °C	74.3 ± 4.36	c	744 ± 1.5	ac
Treatment 45 °C	92.3 ± 4.72	d	665 ± 1.5	ab
Treatment 50 °C	131.7 ± 3.50	e	644 ± 1.2	a

* GAE: gallic acid equivalent. ** AAE: ascorbic acid equivalent. Values are expressed as mean ± SD (*n* = 9). Values followed by the same letter are not statistically different by Duncan’s multiple range test (*p* < 0.05).

## References

[1] (2014). Food and Agriculture Organization of the United Nations, FAOSTAT. http://www.fao.org/faostat/en/#data.

[2] Bertuzzi T., Rastelli S., Pietri A. (2015). *Aspergillus* and *Penicillium toxins* in chestnuts and derived products produced in Italy. Food Control.

[3] Míguelez J.D.L.M., Bernardez M.M., Queijeiro J.G. (2004). Composition of varieties of chestnuts from Galicia (Spain). Food Chem..

[4] Borges O., Gonçalves B., De Carvalho J.L.S., Correia P., Silva A.P. (2008). Nutritional quality of chestnut (*Castanea sativa* Mill.) cultivars from Portugal. Food Chem..

[5] Peña-Méndez E.M., Hernández-Suárez M., Díaz-Romero C., Rodríguez-Rodríguez E. (2008). Characterization of various chestnut cultivars by means of chemometrics approach. Food Chem..

[6] Üstün N., Tosun Y., Serdar U. (1999). Technological properties of chestnut varieties grown in Erfelek district of Sinopy city. Acta Hortic..

[7] Barreira J.C., Casal S., Ferreira I.C., Oliveira M.B.P., Pereira J.A. (2009). Nutritional, fatty acid and triacylglycerol profiles of *Castanea sativa* Mill. cultivars: A compositional and chemometric approach. J. Agric. Food Chem..

[8] Attanasio G., Cinquanta L., Albanese D., Di Matteo M. (2004). Effects of drying temperatures on physico-chemical properties of dried and rehydrated chestnuts (*Castanea sativa*). Food Chem..

[9] Moreira R., Chenlo F., Chaguri L., Vázquez G. (2011). Air drying and colour characteristics of chestnuts pre-submitted to osmotic dehydration with sodium chloride. Food Bioprod. Process..

[10] Nazzaro M., Barbarisi C., La Cara F., Volpe M.G. (2011). Chemical and biochemical characterisation of an IGP ecotype chestnut subjected to different treatments. Food Chem..

[11] Overy D.P., Seifert K.A., Savard M.E., Frisvad J.C. (2003). Spoilage fungi and their mycotoxins in commercially marketed chestnuts. Int. J. Food Microbiol..

[12] Rodrigues P., Venâncio A., Lima N. (2012). Mycobiota and mycotoxins of almonds and chestnuts with special reference to aflatoxins. Food Res. Int..

[13] Prencipe S., Siciliano I., Gatti C., Garibaldi A., Gullino M.L., Botta R., Spadaro D. (2018). Several species of *Penicillium* isolated from chestnut flour processing are pathogenic on fresh chestnuts and produce mycotoxins. Food Microbiol..

[14] Da Rocha M.E.B., Freire F.D.C.O., Maia F.E.F., Guedes M.I.F., Rondina D. (2014). Mycotoxins and their effects on human and animal health. Food Control.

[15] Bennett J.W., Klich M. (2003). Mycotoxins. Clin. Microbiol. Rev..

[16] Marín S., Ramos A.J., Cano-Sancho G., Sanchis V. (2013). Mycotoxins: Occurrence, toxicology, and exposure assessment. Food Chem. Toxicol..

[17] Zain M.E. (2011). Impact of mycotoxins on humans and animals. J. Saudi Chem. Soc..

[18] Kosegarten C.E., Ramírez-Corona N., Mani-López E., Palou E., López-Malo A. (2016). Description of *Aspergillus flavus* growth under the influence of different factors (water activity, incubation temperature, protein and fat concentration, pH, and cinnamon essential oil concentration) by kinetic, probability of growth, and time-to-detection models. Int. J. Food Microbiol..

[19] Spadaro D., Garibaldi A., Gullino M.L., Stack J., Fletcher J., Mumford J. Containment of mycotoxins in the food chain by using decontamination and detoxification techniques. Practical Tools for Plant and Food Biosecurity.

[20] Kabak B., Dobson A.D.W., Var I. (2006). Strategies to prevent mycotoxin contamination of food and animal feed: A review. Crit. Rev. Food Sci. Nutr..

[21] Siciliano I., Spadaro D., Prelle A., Vallauri D., Cavallero M.C., Garibaldi A., Gullino M.L. (2016). Use of Cold Atmospheric Plasma to Detoxify Hazelnuts from Aflatoxins. Toxins.

[22] Siciliano I., Dal Bello B., Zeppa G., Spadaro D., Gullino M.L. (2017). Static hot air and infrared rays roasting are efficient methods for aflatoxin decontamination on hazelnuts. Toxins.

[23] Udomkun P., Wiredu A.N., Nagle M., Müller J., Vanlauwe B., Bandyopadhyay R. (2017). Innovative technologies to manage aflatoxins in foods and feeds and the profitability of application—A review. Food Control.

[24] Pietri A., Rastelli S., Mulazzi A., Bertuzzi T. (2012). Aflatoxins and ochratoxin A in dried chestnuts and chestnut flour produced in Italy. Food Control.

[25] (2013). RASFF. https://webgate.ec.europa.eu/rasff-window/portal/index.cfm?event¼notificationList.

[26] Prencipe S., Siciliano I., Contessa C., Botta R., Garibaldi A., Gullino M.L., Spadaro D. (2018). Characterization of *Aspergillus* section *Flavi* isolated from fresh chestnuts and the chestnut flour process. Food Microbiol..

[27] Commission of the European Communities (2010). Commission Regulation (EC) No 165/2010 of 26 February 2010 amending regulation (EC) No 1881/2006 setting maximum levels for certain contaminants in foodstuffs as regards aflatoxins. Off. J. Eur. Union.

[28] Jermini M., Conedera M., Sieber T.N., Sassella A., Schärer H. (2006). Influence of fruit treatments on perishability during cold storage of sweet chestnuts. J. Sci. Food Agric..

[29] Marín S., Ramos A.J., Sanchis V. (2012). Modelling *Aspergillus flavus* growth and aflatoxins production in pistachio nuts. Food Microbiol..

[30] Liu X., Guan X., Xing F., Lv C., Dai X., Liu Y. (2017). Effect of water activity and temperature on the growth of *Aspergillus flavus*, the expression of aflatoxin biosynthetic genes and aflatoxin production in shelled peanuts. Food Control.

[31] Giorni P., Battilani P., Pietri A., Magan N. (2008). Effect of *aw* and CO_2_ level of *Aspergillus flavus* growth and aflatoxin production in high moisture maize post-harvest. Int. J. Food Microbiol..

[32] Schmidt-Heydt M., Rüfer C.E., Abdel-Hadi A., Magan N., Geisen R. (2010). The production of aflatoxin B1 or G1 by *Aspergillus parasiticus* at various combinations of temperature and water activity is related to the ratio of aflS to aflR expression. Mycotoxin Res..

[33] Gallo A., Solfrizzo M., Epifani F., Panzarini G., Perrone G. (2016). 2016 Effect of temperature and water activity on gene expression and aflatoxin biosynthesis in *Aspergillus flavus* on almond medium. Int. J. Food Microbiol..

[34] Neri L., Dimitri G., Sacchetti G. (2010). Chemical composition and antioxidant activity of cured chestnuts from three sweet chestnut (*Castanea sativa* Mill.) ecotypes from Italy. J. Food Compos. Anal..

[35] Zhu F. (2016). Effect of processing on quality attributes of chestnut. Food Biol. Technol..

[36] Schindler A.P., Palmer J.G., Eisenberg W.V. (1967). Aflatoxin production by *Aspergillus flavus* as related to various temperatures. Appl. Microbiol..

[37] Pitt J.I., Hocking A.D. (1997). Fungi and Food Spoilage.

[38] Magan N., Aldred D., Sanchis B., Arora D.K. (2004). The role of spoilage fungi in seed deterioration. Fungal Biotechnology in Agricultural Food and Environmental Applications.

[39] Lahouar A., Marin S., Crespo-Sempere A., Saïd S., Sanchis V. (2016). Effects of temperature, water activity and incubation time on fungal growth and aflatoxin B1 production by toxinogenic *Aspergillus flavus* isolates on sorghum seeds. Rev. Argent Microbiol..

[40] Trenk H.L., Hartman P.A. (1970). Effects of moisture content and temperature on aflatoxin production in corn. Appl. Microbiol..

[41] Pratiwi C., Rahayu W.P., Lioe H.N., Herawati D., Broto W., Ambarwati S. (2015). The effect of temperature and relative humidity for *Aspergillus flavus* BIO2237 growth and aflatoxin production on soybeans. Int. Food Res. J..

[42] Gonçalves B., Borges O., Costa H.S., Bennett R., Santos M., Silva A.P. (2010). Metabolite composition of chestnut (*Castanea sativa* Mill.) upon cooking: Proximate analysis, fibre, organic acids and phenolics. Food Chem..

[43] Correia P., Leitão A., Beirão-da-Costa M.L. (2009). The effect of drying temperatures on morphological and chemical properties of dried chestnuts flours. J. Food Eng..

[44] Pellegrini N., Searfini M., Salvadore S., Del Rio D., Bianchi M., Brighenti F. (2009). Total antioxidant capacity of spices, dried fruits, nuts, pulses, cereals and sweets consumed in Italy assessed by three different in vitro assays. Mol. Nutr. Food Res..

[45] Vázquez G., Fontenla E., Santos J., Freire M.S., González-Álvarez J., Antorrena G. (2008). Antioxidant activity and phenolic content of chestnut (*Castanea sativa*) shell and eucalyptus (*Eucalyptus globulus*) bark extracts Industrial. Crops Prod..

[46] Galiñanes C., Freire M.S., González-Álvarez J. (2015). Antioxidant activity of phenolic extracts from chestnut fruit and forest industries residues. Eur. J. Wood Prod..

[47] Barros A.I.R.N.A., Nunes F.M., Gonçalves B., Bennett R.N., Silva A.P. (2011). Effect of cooking on total vitamin C contents and antioxidant activity of sweet chestnuts (*Castanea sativa* Mill.). Food Chem..

[48] Ribeiro B., Rangel J., Valentão P., Andrade P.B., Pereira J.A., Bolke H. (2007). Organic acids in two Portuguese chestnut (*Castanea sativa* Miller) varieties. Food Chem..

[49] ISTISAN 1996/34. http://old.iss.it/binary/publ2/cont/Rapporto%2096-34.1140450878.pdf.

[50] 985.29 AOAC. http://www.eoma.aoac.org/methods.

[51] 920.39 AOAC. http://www.eoma.aoac.org/methods.

[52] Singleton V.L., Rossi J.A. (1965). Colorimetry of total phenolics with phosphomolybdic-phosphotungstic acid reagents. Am. J. Enol. Vitic..

[53] Szőllősi R., Szőllősi Varga I. (2002). Total antioxidant power in some species of Labiatae (Adaptation of FRAP method). Acta Biol. Szeged..

